# Unsupervised Hierarchical Clustering Approach for Tourism Market Segmentation Based on Crowdsourced Mobile Phone Data

**DOI:** 10.3390/s18092972

**Published:** 2018-09-06

**Authors:** Jorge Rodríguez, Ivana Semanjski, Sidharta Gautama, Nico Van de Weghe, Daniel Ochoa

**Affiliations:** 1 Department of Telecommunications and Information Processing, Ghent University, St-Pietersnieuwstraat 41, B-9000 Ghent, Belgium; Ivana.Semanjski@UGent.be (I.S.); Sidharta.Gautama@UGent.be (S.G.); 2 ESPOL Polytechnic University, Escuela Superior Politécnica del Litoral, ESPOL, Facultad de Ingeniería en Electricidad y Computación, Campus Gustavo Galindo Km 30.5 Vía Perimetral, P.O. Box 09-01-5863, EC090112 Guayaquil, Ecuador; dochoa@fiec.espol.edu.ec; 3 Department of Geography, Ghent University, Krijgslaan 281 (S8), B-9000 Ghent, Belgium; Nico.VandeWeghe@UGent.be

**Keywords:** tourism management, big data analytics, smartphones, human mobility, behavioural clustering, market segmentation, crowdsourcing

## Abstract

Understanding tourism related behavior and traveling patterns is an essential element of transportation system planning and tourism management at tourism destinations. Traditionally, tourism market segmentation is conducted to recognize tourist’s profiles for which personalized services can be provided. Today, the availability of wearable sensors, such as smartphones, holds the potential to tackle data collection problems of paper-based surveys and deliver relevant mobility data in a timely and cost-effective way. In this paper, we develop and implement a hierarchical clustering approach for smartphone geo-localized data to detect meaningful tourism related market segments. For these segments, we provide detailed insights into their characteristics and related mobility behavior. The applicability of the proposed approach is demonstrated on a use case in the Province of Zeeland in the Netherlands. We collected data from 1505 users during five months using the Zeeland app. The proposed approach resulted in two major clusters and four sub-clusters which we were able to interpret based on their spatio-temporal patterns and the recurrence of their visiting patterns to the region.

## 1. Introduction

People are travelling for many purposes such as going to school, work or shopping, visiting family and friends or going on holidays. Some of these activities, such as tourism related travel, have a strong seasonal character [1]. This affects the mobility conditions at the destination location as well as the traffic network capacities used to reach the desired destinations in an equally seasonal manner. Thus, understanding tourism related mobility behaviour has a strong impact on tourist destination mobility system planning and its reachability [2]. In addition, understanding the patterns that visitors utilize to explore the destination region, whether this is a day visit or a whole vacation period, can serve as a basis for the creation of tourism related activities. The literature shows that provision of the higher level of service at these locations influences the selection of places visited by tourists and the length of their stay in the area [3]. Hence, understanding tourism related mobility behaviour has direct economic implications for the tourist destination regions.

According to the literature, researchers commonly rely on paper-based tourism surveys to collect such information. In order to reach as many travellers as possible, researchers have joined forces with accommodation providers [4,5], international arrival airports or borders control [6,7], and tourist information offices to collect data through quantitative surveys [8,9]. Based on these surveys, the tourist population is commonly segmented in homogeneous groups to which dedicated and personalised services can be provided [4,6,9]. This process is well known as market segmentation and is based on psychographic, socio-economic or activity-choice criteria [10]. Nevertheless, conducting paper-based surveys exhibits several disadvantages as failing to capture longitudinal behaviour (e.g., do visitors return to the study area, the frequency of these returns). Keeping in mind that the knowledge about the guest in all phases of the journey is essential for the tourist sector, the application of wearable sensors seems a promising line of research to complement, or even replace, time and budget demanding paper-based surveys. Smartphones can be considered as one of these sensors, being worn voluntarily by individuals on a daily basis and capable of collecting data via a bundle of integrated sensors such as GNSS (Global Navigation Satellite Systems), cameras, microphones, accelerometers and many more. In addition, they provide two-way communication and can be used for completing digital surveys instead of the paper-based ones. To operationalize this, big data analytics become an important factor as processing this crowdsourced data requires dedicated algorithms capable of extracting meaningful insights. Literature shows great interest in this domain over the past years, highlighting the importance and the added value of crowdsourced data in the decision-making and planning processes [11,12,13,14,15,16,17]. Although some work has already been done in the field of big data applications for tourism [18,19,20], the potential of the tourism market segmentation based solely on the ubiquitous massive sensing using tracking data obtained by smartphones is still unexplored.

Extending the current research in the tourism management domain, and in the meantime addressing the above-mentioned limitations, the aim of this study is to explore how ubiquitous massive sensing systems can be used in tourism population segmentation and what additional tourism mobility related insights can be extracted based on such approach. In order to achieve this, we collected data from over 1500 participants, over a period of five months, in the touristic Province of Zeeland, the Netherlands. We have developed a hierarchical clustering approach to extract tourism market segments and give detailed insight into mobility behaviour of each of these segments. In more detail, the fundamental research contributions of this work can be situated in the following areas: (i) we demonstrate the applicability of the ubiquitous massive sensing for tourism management purposes and related decision-making processes; (ii) we develop a big data based market segmentation approach for tourism applications; and (iii) we provide the first big data based insight into mobility behaviour of different tourism market segments.

The remainder of this paper is organized as follows: Section 2 gives an overview of the research area and the collected data. This section also includes a detailed description of the method applied to detect and interpret the tourism clusters (market segments). The results are given in Section 3, followed by the discussion of the findings and concluding remarks in Section 4.

## 2. Materials and Methods

As already mentioned, the paper presents an analysis of crowdsourced data collected by means of smartphones in the Zeeland province, the Netherlands. We have used the mobile application called Zeeland to collect the data during the tourism mobility campaign called Traffic in Zeeland.

### 2.1. Study Area

In 2014, 12.5 million Dutch people went on holidays. More than half of them had a domestic destination [21], making the watersports regions and the North Sea beach resorts the most popular national tourist destinations. The main transport mode for these trips, within the country or abroad, is cars. It is reported that, in 9 out of 10 cases, Dutch people use cars to reach their holiday destinations [21]. Even though in almost all Dutch provinces traffic is much less dense between July and August, since there is less commuter traffic, the Zeeland province is the only one where the traffic density is higher during this period in comparison with the other months, due to the large amount of visitors.

**Figure 1:**
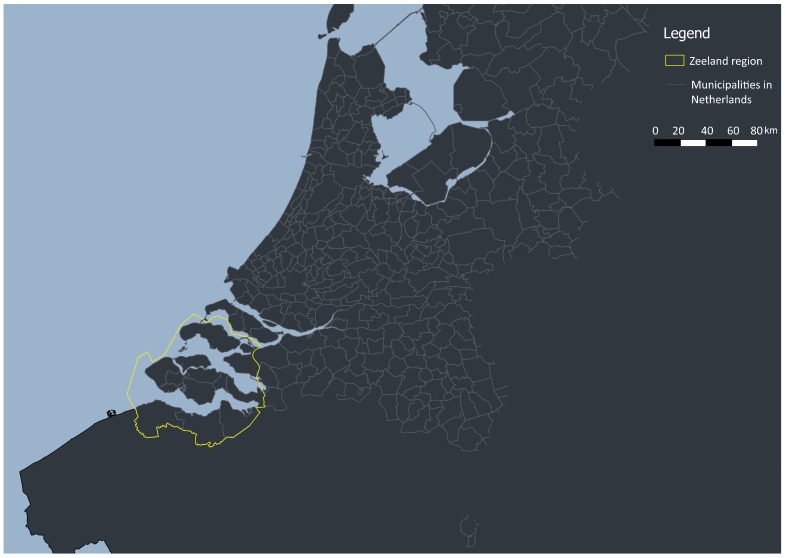
Province of Zeeland, the Netherlands.

Geographically, Zeeland is situated in the southwest of the Netherlands and includes about 2930 square kilometers area composed of of shores and islands (Figure 1). Overall, around 380,000 people live in this region. With respect to the total employment in the province, 9.4% come from the touristic activities, representing more than 16,000 jobs. Moreover, according to the Statistics Netherlands, a Dutch governmental institution, known in Dutch as Centraal Bureau voor de Statistiek (CBS), the total number of tourist overnight stays in 2016 in Zeeland is estimated to be 15 million. Hence, the economic importance of the tourism in this province raises the necessity to better understand the behaviour of its visitors and their mobility patterns. The province is well connected with the rest of Europe. The tourists enter this region by using bicycle, ferry, bus or train. To reach the area by plane, they need to use one of the airports situated outside of the province and continue their trip towards the final destination by one of the above-mentioned transportation modes.

### 2.2. Mobile Application and Data Collection

**Figure 2:**
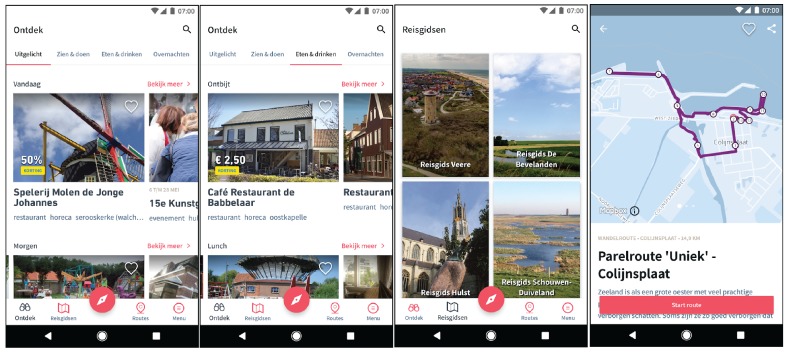
Screenshots of the Zeeland mobile application.

The data used for this study was collected by the Zeeland mobile application (Figure 2), provided by the official regional tourism information agency VVV Zeeland (Province of Zeeland, the Netherlands). This application offers information services for the Zeeland visitor and, at the same time, asks the user to share data for tourism insights. Once installed on a smartphone, it starts logging data using the sensors present in the smartphone, i.e., GNSS receiver. The data logging uses technology from Sentiance (Antwerp, Belgium), a company specialized in Internet of Things (IoT) and data science technologies. Sentiance technology offers contextual insight into consumer behaviour by documenting the transport modes and the purpose of travelled trips.

### 2.3. Dataset

**Figure 3:**
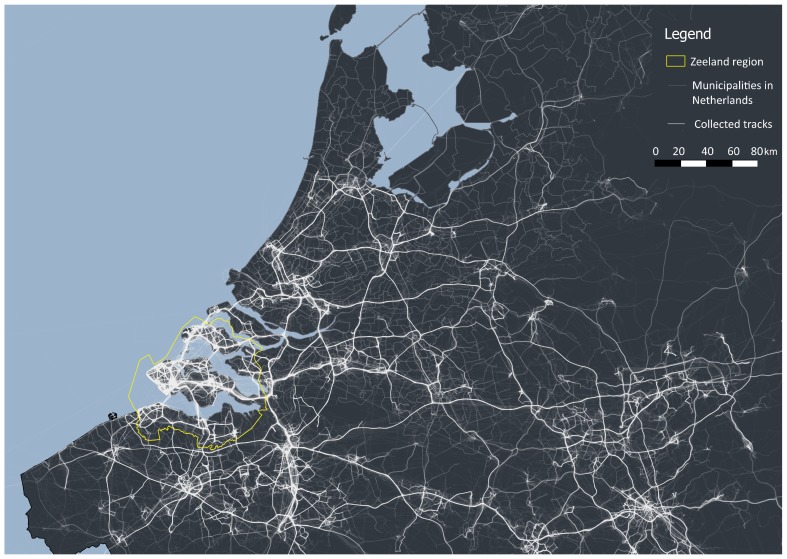
Data collected by the Zeeland mobile application.

The data collection process in this study lasted from May to September 2017. During this period, a total of 10,597 users downloaded the application of which 1,505 users contributed their data. This resulted in the collection of 124,725 trips and 151,612 trip segments, where a trip represents user movement between the trip’s origin and destination location, while a trip segment represents a part of the trip where a single transport mode is used. Figure 3 illustrates the collected dataset with the indication of the study region.

In our dataset, each record represents a trip segment. A detailed description of attributes collected for each trip segment is given in Table 1.

### 2.4. Cluster Analysis

This study focuses on understanding Zeeland visitors’ mobility behaviour. Thus, we aimed to identify and characterize the different tourist segments according to their visiting pattern. However, this is data of unknown structure. Unsupervised learning techniques are able to explore data structures in order to extract meaningful information [22]. Therefore, in order to achieve our goal, we have used unsupervised clustering.

First, a data cleaning process was performed. Trip segments with missing data or empty fields were excluded. Using the valid trip segments, we established whether the trip segment’s start and end points were inside or outside the study region. A user’s trip segments were sorted by start time and aggregated to create a trip diary summary. The aim was to discover the periods when the user is “outside”, “going to”, “inside” and “leaving” the study area. It should be noted that users who visit the study area frequently can exhibit this visiting pattern several times. On the other hand, users who did not leave the study area will only have one record labelled as “inside”, while users who did not visit the study area during the campaign will have one record labelled as “outside”. Finally, an object to represent the travelling characteristics of each user was created based on his trip diary summary. This object is made of the variables showed in Table 2.

Clustering techniques cannot handle objects with heterogeneous types of variables: numerical and categorical (non-numerical value) [23,24]. Categorical variables with two levels can be represented as binary variables which are still discrete and can be handled by machine learning algorithms such as clustering techniques [25]. Categorical variables with more than two levels are not possible to use because distance or similarity metrics cannot be computed directly. In our case, the stay variable is a categorical variable with five levels. We solved this problem using the one-hot encoding technique to map the categorical variable to an *n*-dimensional space. The idea behind this approach is to create a *n* new dummy variables according with the *n* levels (values) of the categorical variable [26]. We converted the stay variable into five new variables: *stay_0, stay_1, stay_2, stay_3, stay_4*. The mapping between the stay variable categories and the dummy variable values is shown as follows:

(1) A:{10000},B:{01000},C:{00100},D:{00010},E:{00001}.

An object is now represented by a seven-dimensional space which allows us to calculate the Euclidean distance.

After characterizing the behaviour of each user, the challenge was to discover similarities among them. Clustering creates groups, or clusters, based on how “similar” or nearby objects are into the dataset. This means that objects belonging to a given cluster are similar to each other rather than objects in other clusters. This requirement seems quite comparable with the definition of market segments who should be as homogeneous (“similar”) as possible within the segment and as heterogeneous (“dissimilar”) as possible when compared to other market segments.

For our clustering approach, similarity is defined in terms of how “close” the objects are to each-other in an *n*-dimensional space. A similarity function allows us to measure the similarity between the properties of two objects, in this case between two tourists. The similarity function for *i* and *j* tourists returns the value *0* when their properties are unalike. On the other hand, a value of *1* means that their properties are identical, which is known as a complete similarity. Hence, the higher the similarity value, the greater the similarity among objects [27]. The similarity measure implemented in our model is the Euclidean distance between two objects (Equation (2)). This is defined as follows:

(2) $$d\left(i,j\right)=\sqrt{{\left(x_{i1}-x_{j1}\right)}^{2}+{\left(x_{i2}-x_{j2}\right)}^{2}+\ldots +{\left(x_{ip}-x_{jn}\right)}^{2}},$$

where $i=\left(x_{i1},\;x_{i2},\;\ldots ,\;x_{in}\right)$ and $j=\left(x_{j1},\;x_{j2},\;\ldots ,\;x_{jn}\right)$ represent two objects described by *n* numeric attributes.

In this study, we are interested in how tourists can be partitioned into groups and at the same time we want to know whether or not there were different levels of aggregation. Because of this, we used a hierarchical method by creating a hierarchical decomposition of the given set of data objects [27]. We used a bottom-up strategy which starts with each object forming a separate group. This approach is known as agglomerative clustering.

The method starts with *n* clusters, one object per cluster, and then iteratively merges existing clusters into larger and larger clusters, depending on how close they are to one another. For example, the initial clusters made up of just a and just b are merged into a new cluster ab. Then, the process that continues searching for the clusters that are closest to one another is performed to merge them, and so on [28]. At the end, the last cluster is composed by all the objects in the dataset, representing the root of the hierarchy. Clusters are merged according to a similarity (linkage) measure, i.e., a distance metric. We used the maximum Euclidean distance (Equation (3)), to measure the distance between two clusters and to decide whether they will be linked or not. This similarity function is defined as follows:

(3) $$distmax\left(C_{i},C_{j}\right)=\max_{p\in C_{i},p^{\prime }\in C_{j}}|p-p^{\prime }|,$$

where *p* represents an object from the cluster $C_{i}$, *p*′ is an object of cluster $C_{j}$, and |*p* − *p*′| represents the distance between both objects.

**Figure 4:**
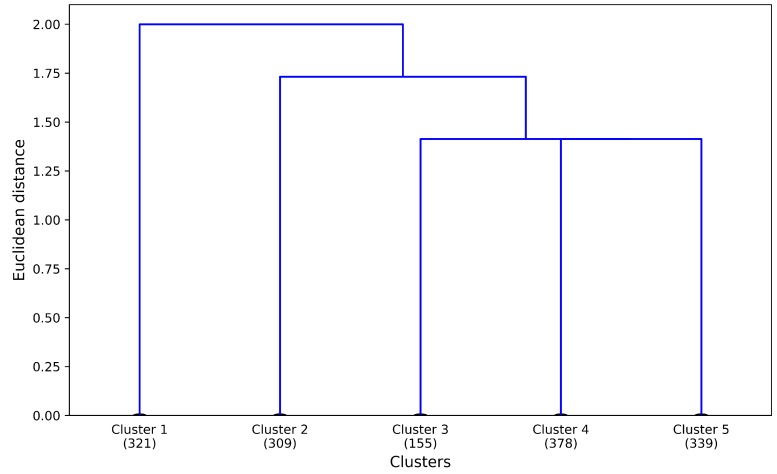
Tourist segments in Zeeland.

Finally, we used a dendrogram to represent the process of hierarchical clustering. We are able to identify a given clustering plotting a horizontal line on the dendrogram at any level. This means that, if we move the horizontal line to the bottom, we will get *n* clusters with one single object in each one. On the other hand, moving the horizontal line to the top will result in one cluster which contains *n* objects. Therefore, we could change the granularity of the abstraction level by cutting the dendrogram through any horizontal line [28]. Nevertheless, the optimal number of clusters still needs to be determined, which will be explained in the following section. The result of this first stage of the hierarchical clustering method is shown in Figure 4.

### 2.5. Cluster Quality for Cluster Selection

In the second stage of the method, we use cluster quality as an indicator to determine the optimal number of clusters. A clustering outcome can be assessed by four criteria: compactness, isolation, global fit, and intrinsic dimensionality [29]. There are many metrics to quantify the quality of the outcomes from an unsupervised clustering technique [30]. We measured the quality of the different aggregation levels in our dendrogram through a silhouette analysis, a graphical tool to plot the silhouette coefficient, which is an average of the ratio of each cluster’s compactness and isolation with range (−1, 1) [31]. The compactness criterion measures the uniqueness of an individual cluster with respect to its environment, while the isolation criterion measures the separation between a cluster and its environment. The silhouette coefficient is defined as follows:

(4) $$S^{\left(i\right)}=\frac{b^{\left(i\right)}-a^{\left(i\right)}}{\max\left\{b^{\left(i\right)},a^{\left(i\right)}\right\}},$$

where $a^{\left(i\right)}$ represents the cluster compactness that is calculated as the average distance between a sample $x^{\left(i\right)}$ and all other points in the same cluster, and $b^{\left(i\right)}$ represents the cluster isolation that is calculated as the average distance between the sample $x^{\left(i\right)}$ and all samples in the nearest cluster.

For each concrete clustering identified on the dendrogram after plotting horizontal lines *h*, we computed the average of the silhouette coefficient of the clusters. Finally, we selected the clustering with the maximum average silhouette coefficient. This metric is suitable to evaluate cluster quality because it does not require the computation of cluster centers and any distance metric can be used.

## 3. Results

This section presents results of our clustering analysis to discover the distinguishing tourist segments. Moreover, we present insights about their mobility behaviour.

### 3.1. Cluster Selection

The dendrogram depicted in Figure 4 shows clusters of users at different abstraction levels. Two clusters were identified at *h* = 2.00. If we cut at *h* = 1.73, three clusters were identified. Finally, five clusters were identified when we cut at *h* = 1.41.

**Figure 5:**
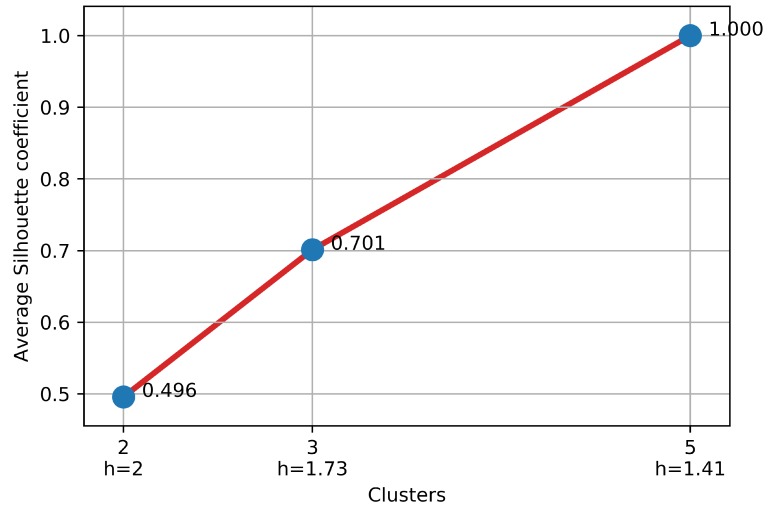
Average silhouette coefficient by clusters identified.

As mentioned before, the optimal number of clusters was selected based on the average silhouette coefficient. Figure 5 shows the results of this metric applied for every clustering identified. We selected the five clusters identified at *h* = 1.41 because they have the highest average silhouette coefficient, so the clustering with the best quality.

**Figure 6:**
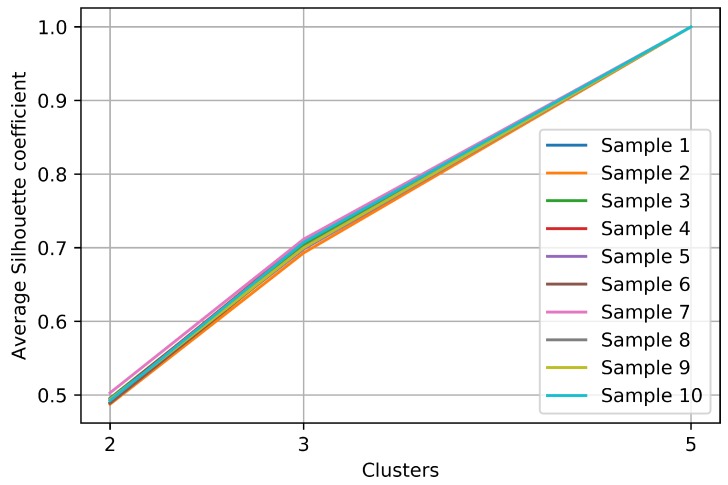
Silhouette coefficient by sampling replication.

In this work, the analysis of the crowdsourced tourism data is data-driven, i.e., unsupervised clustering has been used to make explicit the inherent structure in the data. This means that there is no application ground truth data to explicitly validate the above results. However, to explore the stability of the process, we replicated our experiment 10 times. For each replicate, we selected randomly 1000 users to perform the same clustering analysis. Results show that the process is stable. Figure 6 shows the quality of every clustering identified in each replicate.

### 3.2. Tourist Segments

In this section, we characterize the clusters (tourist segments) identified when cutting the dendrogram at *h* = 1.41. These tourist segments have the following characteristics:

- **External unsorted (Cluster 1)**. This sub-cluster aggregates users for whom trip segments were registered, but none of them were in the Zeeland region during the study period.
- **Internal (Cluster 2)** users correspond to those users for whom multiple trips were observed and they all both started and ended within the Zeeland region. There were no trips noted outside the Zeeland region nor any of them crossed the outer borders of the Zeeland region. The interpretation of internal users might be two-fold. On one end, they might be local residents who use the app. On the other end, they might be external visitors who started using the app after they already arrived within the Zeeland region and they also might have uninstalled the app before leaving the Zeeland region, making all of their observed trips limited to the duration of the staying period within the region.
- **External 24 (Cluster 3)** captures the moving patterns where only one entry in the trip diary summary has been labelled as “inside”. However, the trip diary summary registered more entries which were labelled as “outside”, “going to” or “leaving” with respect to the Zeeland region. Furthermore, the time period of the “inside” observation is less than 24 h. This cluster contributes to the tourism class “day tourist”.
- **External long (Cluster 4)**. This sub-cluster captures the moving patterns where only one entry in the trip diary summary has been labelled as “inside” the Zeeland region. Nevertheless, the time period between the entry’s start and end trips is longer than 24 h. This cluster contributes to the tourism class “longer-stay tourist”.
- **External recurring (Cluster 5)** captures the moving patterns where multiple entries in the trip-diary were labelled as “inside” the Zeeland region. Moreover, those entries are interspersed with entries labelled as “outside”, “going to” and “leaving” the Zeeland region, which means that the user enters and leaves the region frequently. For users within this sub-cluster, no distinction was made based on the observed time period that passed between sequential trips with altering Zeeland region as trip’s origin or destination (meaning that one user can have a mixture of staying time periods both shorter and longer than 24 h).

**Figure 7:**
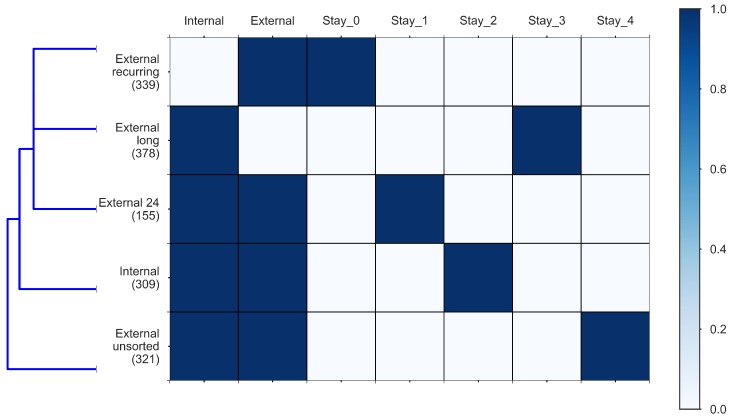
Heat map of tourist segments vs. dataset features.

We see in Figure 4 that tourist segments “External long”, “External 24” and “External recurring” are the most similar ones based on the Euclidean distance metric. Figure 7 depicts through a heat map the relation between the identified tourist segments and the used variables.

### 3.3. Transport Mode Insights

**Figure 8:**
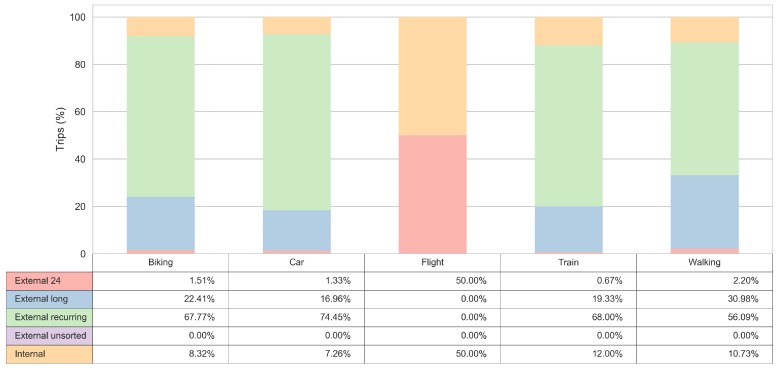
Tourist behaviour by transport mode.

The frequency of the matched sub-cluster labels for the visitors and the observed transport modes for the trip segments is shown in Figure 8. The interpretation of the sub-clusters distribution for trips made by transport mode flight is limited by the small sample of users who utilized these transport modes; nonetheless, a better insight can be gained for other transport modes.

**Figure 9:**
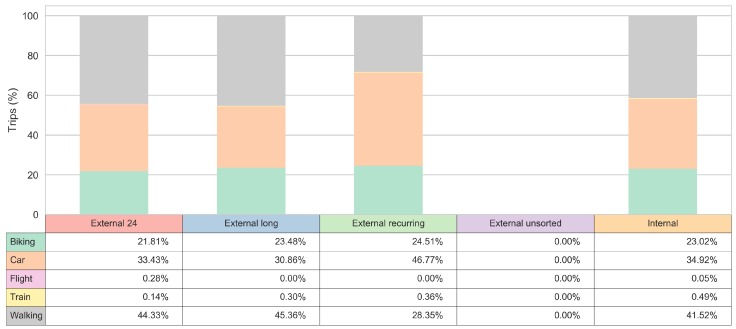
Transport mode choices by tourist segment.

Figure 9 reveals that the ’External long’, ’External 24’, and ’Internal’ clusters are more prone to take a walk. The “External recurring” sub-cluster has a quite high number of car related trips, exhibiting an almost equal share pattern with “External long” sub-cluster when it comes to the use of bikes. The lowest bike usage is present in ’External 24’ sub-cluster.

### 3.4. Distance Travelled Insights

Discovering how far tourists and citizens travel in a region is important to establish traffic management strategies such as enhancing public transport, promoting routes, network improvement, among others [32]. In this section, we took a look about the distance travelled for every tourist segment.

**Figure 10:**
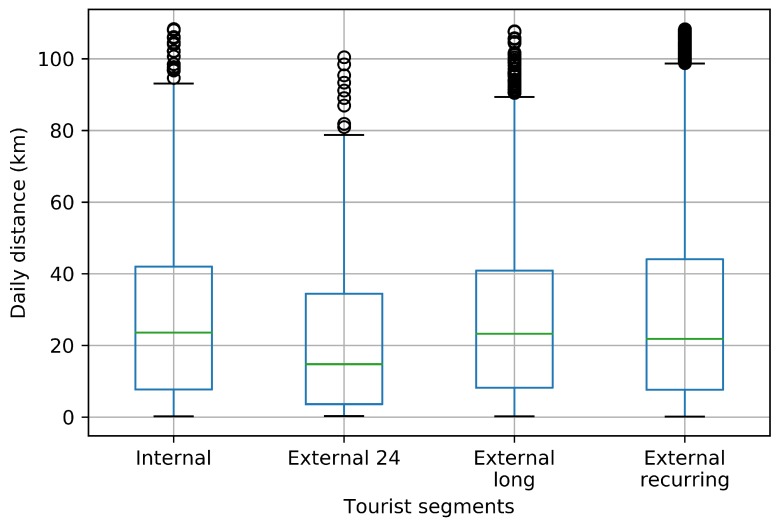
Daily distance travelled.

Figure 10 depicts the daily distance travelled by each tourism segment. The ’External 24’ cluster has the lowest maximum distance travelled per day due to the users inside this cluster visiting the region only for a few hours. This figure also reveals that the ’Internal’, ’External long’ and ’External recurring’ clusters exhibit a similar daily distance travelled behaviour.

**Figure 11:**
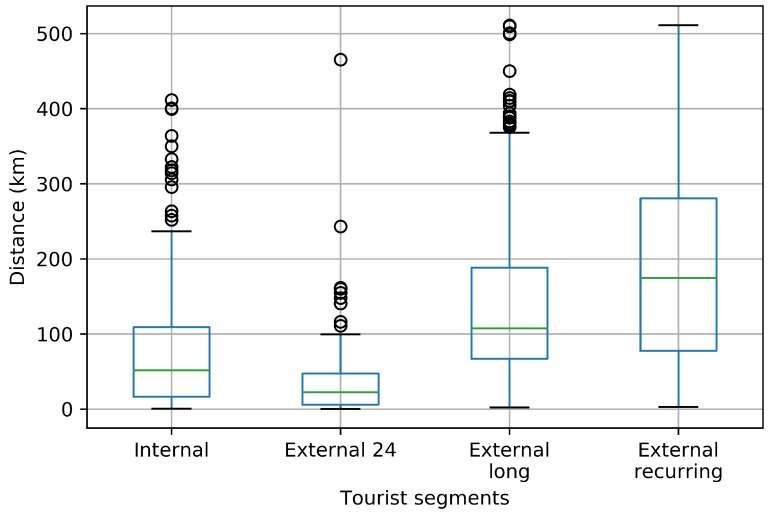
Total distance travelled.

The total distance travelled for every tourist segment is shown in Figure 11. The ’External 24’ cluster shows the same behaviour observed in Figure 10 because the users who belong to this cluster are daily visitors. The ’Internal’ cluster represents the residents of the area. These users could have installed the application for a short period of time, thus it was not possible to register longer distances travelled. The ’External long’ and ’External recurring’ clusters register the longest distances travelled.

### 3.5. Trip Origin Locations

**Figure 12:**
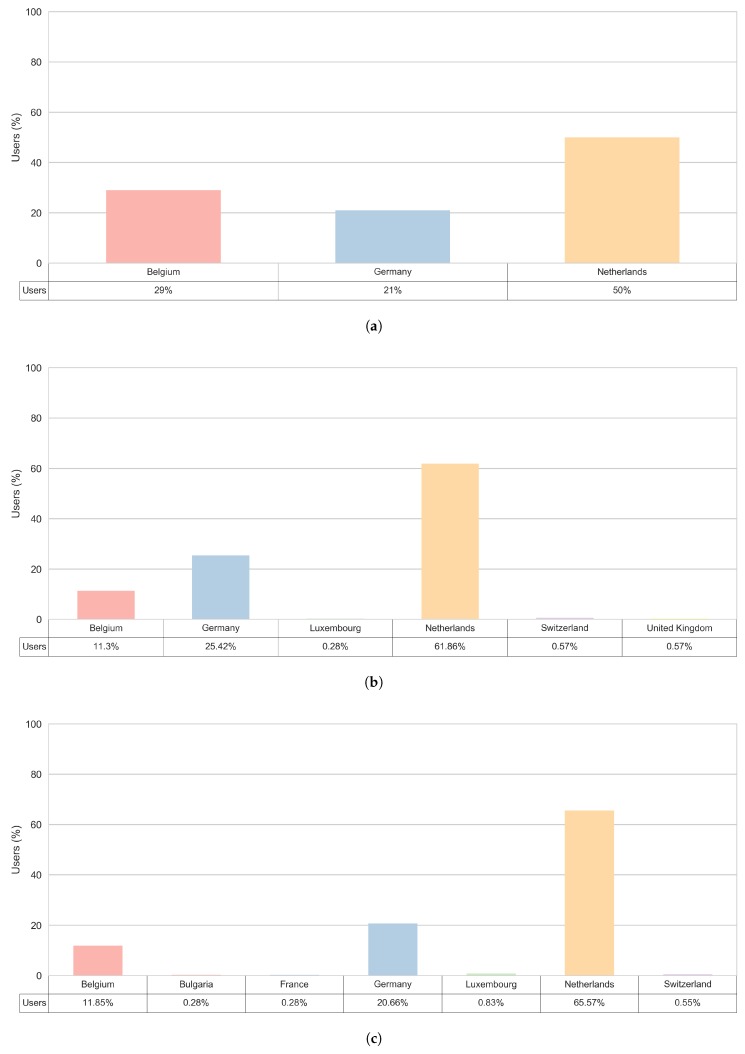
Country of the origin for trips that end in the Zeeland region. (**a**) External 24; (**b**) External long; (**c**) External recurring.

In order to gain a better insight into potential tourism related trips, we took a deeper look at the tourist clusters and the distribution of the trips’ origins for trips that end within the Zeeland region. Figure 12 shows the distribution of external visitors by country where they come from. Notably, all three sub-clusters observed within the Zeeland region have three main countries of origin for trips that end in the Zeeland region and these are:

1. The Netherlands—always the country with the most trips’ origins, for each external user’s sub-cluster,
2. Germany—the second most frequent country of origin, for all external users’ sub-clusters except for the ’External 24’,
3. Belgium—the second most frequent country of origin for the ’External 24’ sub-cluster and the third most often country of origin for other external users’ sub-clusters.

Figure 12a gives an overview of the estimated home origin of the users in the corresponding ’External 24’ sub-cluster, i.e., the users that spent a day in the Zeeland region. The most of these trips come from within the Zeeland region and other provinces in the Netherlands. Next to the trips that originated in the Netherlands, most of the observed trips originated in Belgium, in the Antwerp and the Ghent area. The trips that originated in Germany were mainly from the municipalities Cologne, Monchengladbach and Oberhausen.

Other countries that appear as the origin locations for the trips that end within the Zeeland region are: Bulgaria, France, Luxemburg, Switzerland and United Kingdom. Furthermore, the most diverse set of the origin countries is noted for the ’External recurring’ sub-cluster. This is shown in Figure 12c.

## 4. Discussion

Considering the application of smartphone sensed data for tourism related market segmentation, we have have found that the hierarchical clustering approach can be successfully applied to segment tourist population into homogeneous and meaningful groups (clusters). Furthermore, mobile sensed data have the potential to support longitudinal analysis of tourist behaviour (as detection of returning tourists and day visitors) and thus overcome the lacks of the traditional paper-based tourism surveys. In addition, data collected in this way exhibit high coverage of the population in an economically neutral way when compared to the surveys, which, for example, collect data at the international airports and thus cover only those tourists who can afford this mode of travelling to the final destination.

In this paper, we used data collected from 1505 app users, which recorded 124,725 trips and 151,612 trip segments. Processing such data in a way that spatial intelligence (awareness of the study area geographical borders), temporal analysis (duration of a stay in this area) and detection of the visiting pattern characteristics (frequency of trips to the target area) are considered can contribute to identification of different tourism market segments. Based on the analysis of these segments, the presence of two main user clusters within the dataset was observed. An ’External unsorted’ user cluster that envelops all the users for whom only trips outside the Zeeland region were observed and a user’s cluster that envelops other users for whom at least one trip was observed into the Zeeland region. This last users cluster is further divided into four sub-clusters: ’Internal’ user cluster that envelops all the users for whom only trips within the Zeeland region were observed; ’External 24’ sub-cluster, those who spent less than 24 h in the Zeeland region in only one occasion; ’External long’, those who spent longer than 24 h in the Zeeland region in only one occasion; and ’External recurring’, those for whom multiple trips in and out of the Zeeland region were observed

Considering these mobile sensed clusters, a high level of similarity to what is traditionally known as tourists, returning tourists and day tourists can be observed. Furthermore, similarity between the smartphone sensed most common origin countries for the visitors to the Zeeland area and the statistics on the most common international visitors to the Netherlands [33] indicate that the smartphone data have the potential to successfully represent the tourism population in a given area as well as to provide more longitudinal insights into tourism related mobility behaviour.

For each of the observed clusters (market segments), we give a detailed insight into their observed mobility behaviour. Some of the results indicate that the ’External long’ and the ’Internal’ user clusters are more prone to take a walk. The other two sub-clusters, although they both have a quite high share of car related trips, exhibit different pattern when it comes to the use of bikes. Finally, the ’External recurring’ sub-cluster has almost an equal share of bike usage as the ’External long’ and the ’Internal’ clusters, while the bike share is the lowest for the ’External 24’ sub-cluster.

The view of the relative number of car trips within the Zeeland region strongly highlighted that, in some municipalities, cars are mainly used for the regional travels. Moreover, the majority of visitors come from the Netherlands. This finding seems to be in line with the results of the national report from Statistics Netherland [21], which states that a car is the most common mean of transportation used by Dutch people when travelling to holidays.

An important consideration for organizations to take into account in this work is data protection and privacy of individuals. For instance, the General Data Protection Regulation (GDPR) is a European Union (EU) regulation that requires businesses to protect the personal data and privacy of EU citizens for transactions that occur within EU member states. Data gathering as performed within the Zeeland app falls under these rules. It does not prohibit data gathering, but it puts important conditions on it like transparency, data minimization, purpose and storage limitation [34]. Within the Zeeland campaign, care was taken to fully comply to these rules.

Potential limitations of such a smartphone sensed data based approach for tourism market segmentation can be seen as the lack of socio-economic and psychographic descriptors in the clustering process. Indeed, such details are not explicitly available in the mobile sensed data; however, implementation of the two-way communication channel over the smartphone can allow collection of such data via, for example, digital survey. Although this might present a certain burden to the tourists who, in return, might be unwilling to complete such surveys during holidays. Alternatively, additional data processing can be applied to try to extract insights on these aspects from the smartphone sensed data, where some examples in the literature in this domain already exist [35]. Another line of research might be focused on implementation of more complex similarity measures in the clustering approach and more detailed spatial intelligence based predictors as visiting expensive shopping areas and restaurants or enjoying long nights out and sport activities, for example.

Following this, the future lines of research will be focused on gaining insight into willingness of tourist population to provide additional information via a mobile application integrated survey and comparison of these results with the extended clustering approach, which would focus on the potential to extract socio-economic and psychographic descriptors from the smartphone sensed data in a non-intrusive way.

## 5. Conclusions

In this paper, we have explored the potential to use the hierarchical clustering approach to detect meaningful clusters of tourism mobility behaviour from smartphone sensed data. The motivation for this lies in the traditionally applied tourism market segmentation that has a strong implications for the provision of personalised tourism services as well as the provision of a higher level of service to these visitors. The obtained results indicate a strong potential for smartphone sensed data to replace traditional, paper-based, tourism surveys in a seamless and less time and resources demanding way. However, several questions still remain for future research and these are mainly focused on obtaining additional (socio-economic and psychographic) insights.

## Figures and Tables

**Table 1:** Description of the variables.

Variable	Acronym	Description
User’s ID	userid	Unique identifier of the user.
Start time	start	Timestamp when the trip segment started.
End time	end	Timestamp when the trip segment ended.
Mode of transportation	mode	Mode of transportation used in the trip segment.
Distance	distance	Distance traveled between the trip segment’s starting and ending points measured in meters.
Waypoints	waypoints	Trajectory of geographic locations (latitude, longitude) followed from the trip segment’s starting until ending point. Additionally, every geography location contains the timestamp when the measure was gathered.
Duration	duration	Duration of the trip segment measured in seconds.

**Table 2:** Description of the variables for the clustering process.

Variable	Acronym	Description
Internal trips	internal	Represents whether or not the user has trip segments into the study region.
External trips	external	Represents whether or not the user has trip segments out the study region.
Staying period	stay	Represents the staying period of the user inside the study area: none-stay (A), one visit of less than 24 h (B), one visit of more than 24 h (C), do not leave the study area (D), recurrent visits of any amount of hours (E).
